# Development of a Hydroxypropyl-β-Cyclodextrin-Based Liquid Formulation for the Oral Administration of Propranolol in Pediatric Therapy

**DOI:** 10.3390/pharmaceutics15092217

**Published:** 2023-08-27

**Authors:** Marzia Cirri, Paola Mura, Simona Benedetti, Susanna Buratti

**Affiliations:** 1 Department of Chemistry Ugo Schiff (DICUS), University of Florence, 50019 Sesto Fiorentino, Italy; paola.mura@unifi.it; 2 Department of Food, Environmental and Nutritional Sciences (DeFENS), University of Milan, 20133 Milan, Italy; simona.benedetti@unimi.it (S.B.); susanna.buratti@unimi.it (S.B.)

**Keywords:** propranolol, pediatric solutions, hydroxypropyl-β-cyclodextrin, photo stability, storage stability, electronic tongue

## Abstract

Propranolol (PPN) is widely used in children to treat various cardiovascular diseases. The availability of a suitable PPN solution should avoid recourse to extemporaneous preparations of unknown/limited stability, as commonly made in hospital pharmacies. However, the development of pediatric PPN solutions is hindered by their instability to light and stability at pH ≈ 3, bitter taste, and the need to improve palatability and avoid co-solvents, flavoring agents, or preservatives that are potentially toxic. In this study, cyclodextrin (CD) complexation has been exploited to develop a safe, stable, and palatable oral pediatric solution of PPN. An initial screening among various CDs allowed us to select HPβCD for its good complexing ability and no toxicity. Drug-HPβCD physical mixtures or co-ground systems (1:1 or 1:2 mol:mol) were used to prepare 0.2% *w*/*v* drug solutions. Photo stability studies evidenced the protective effect of HPβCD, revealing a reduction of up to 75% in the drug degradation rate after 1 h of exposure to UV radiation. Storage stability studies showed unchanged physical–chemical properties and almost constant drug concentration after 6 months and under accelerated conditions (40 °C), despite the less aggressive pH (≈5.5) of the solution. The electronic tongue test proved that the HPβCD taste-masking properties improved the formulation palatability, with a 30% reduction in drug bitterness.

## 1. Introduction

One of the major problems in pediatric medicine is the lack of suitable specific age-appropriate formulations. This has, as a consequence, the frequent off-label and unlicensed drug use, with the recourse to extemporaneous preparations enabling to administer the required dose and/or facilitate medicine administration [1,2,3]. These preparations are obtained by manipulations of adult dosage forms, mainly by tablet dividing and crushing or the opening of capsules and dispersing the powders in liquids [4,5]. However, these manipulations can give rise to problems of a loss of dosing accuracy and content uniformity, a lack of information about the physical, chemical, and microbial stability of the preparation, and changed/unknown bioavailability. Furthermore, the obtained products can result unpleasant in taste and appearance, leading to poor compliance in the pediatric population [6,7]. Moreover, the presence in such formulations of excipients such as preservatives, colorants, sweeteners, flavoring agents, or organic solvents, which could be potentially harmful to children, particularly for infants and neonates, should be considered [8,9,10,11]. In fact, safety concerns with some excipients may emerge when they are used in products intended for the pediatric population, particularly in infants [12], as has been reported, for example, for parabens [13,14], benzalkonium chloride [14,15], saccharin sodium [14,15], propylene glycol [14,16,17], and ethanol [14,18]. Safety concerns associated with flavoring agents regarding potential risks of allergy and sensitization have also been reported, despite the fact that they are commonly used in smaller amounts than other excipients [18]. A review of the worldwide current state of pharmaceutical excipients in pediatrics has been recently published [19].

The ideal oral pediatric formulation would allow flexible dosing, be easy to administer and palatable, have suitable physical and microbiological stability, and contain only excipients that are safe for children [20].

Among the different dosage forms, liquid formulations are considered the best choice for the pediatric population, as also reported by the European Medicinal Agency [21], since they are very simple and quick to administer and swallow and allow easy dosage adjustments [22,23]. Among the liquid formulations, solutions are preferred to suspensions, which present higher problems of poor physical stability with possible caking phenomena and/or difficulties in achieving a homogeneous re-dispersion of the sediment, with consequent poor reproducible dosing due to the potential for dose withdrawal heterogeneity. However, liquid formulations present some drawbacks with respect to solid dosage forms that have to be taken into consideration in their design, such as, in particular, their greater issues of stability and palatability being the last crucial parameter to achieve good compliance and adherence to the therapy of pediatric patients [18,23].

Propranolol is a non-selective β-blocker that is widely used in children for the treatment of various cardiovascular diseases, including hypertension and different kinds of arrhythmias, and it can be also used to treat migraine headaches [24]. Moreover, it is now considered the first-line therapy in the treatment of infantile hemangiomas [25]. Due to its poor water solubility (0.07 g/L), it is commonly commercialized as hydrochloride salt (solubility 10 g/L). In addition to its limited solubility, other drawbacks limiting the development of stable and effective propranolol solutions are related to their instability to light [26], their unpleasant bitter taste [26], and their maximum stability in solution at pH 3, although they rapidly decompose at alkaline pH with discoloration and pH reduction [27].

In Italy, propranolol liquid formulations intended for pediatric use are commonly extemporaneously prepared by hospital pharmacies according to the instructions of the galenic Handbook of SIFO (Società Italiana Farmacie Ospedaliere) [28] by dissolving the propranolol hydrochloride salt in a sucrose-based syrup containing citric acid as a stabilizer and raspberry flavor as a taste-masking agent. However, no clear data are given about the stability of these extemporaneous preparations, whose indicated expiration dates vary from 30 to 60 days. Moreover, the presence of sucrose makes them unsuitable for diabetic patients. Furthermore, a pH of around 3, obtained by adding citric acid, is necessary to improve drug stability but can reduce patient acceptability. Finally, safety concerns may regard the pediatric use of the raspberry flavor, which is added to masque the drug’s bitter taste [18]. On the other hand, the only commercially available pediatric solution of propranolol hydrochloride that is specifically intended for the treatment of infantile hemangiomas, in addition to being very expensive, contains citric acid as a stabilizer (pH around 3), and some excipients, such as sodium saccharin as a sweetener, strawberry, and vanilla as flavoring agents, and propylene glycol (2.6 mg/mL) as a solvent, whose daily intake should be limited since they are all considered potentially harmful in pediatrics and have been associated with toxicity, particularly in neonates [14]. Moreover, additional effects in the daily intake dose calculation should also be considered due to the large use of these same excipients as food additives.

Although its primary and effective use is to enhance the aqueous solubility of poorly soluble drugs [29,30,31], cyclodextrin (CD) complexation proved to be an interesting tool to mask the unpleasant taste of drugs [32,33,34,35,36,37,38], as well as improve their chemical stability [3,39,40,41], including photostability [42,43,44,45]. The excellent safety profile of orally administered CDs [29] when considering their very low oral bioavailability (0.1–3%) [46] make them valuable excipients in pediatric formulations.

Based on these premises, the aim of this study was to evaluate the possibility of exploiting the solubilizing, stabilizing, and taste-masking properties of CDs to develop a safe, stable, acceptable, and palatable liquid formulation (0.2% *w*/*v*) of propranolol hydrochloride aimed for oral administration to pediatric patients. The proposed formulation would allow us to avoid the use of organic co-solvents, acidifiers, sweeteners, and flavoring agents (and all their possible toxicity problems) and respect the requirement for using minimum excipients so as to reduce possible safety concerns and incompatibility issues.

An initial screening was carried out in order to select the most effective CD able to complex the drug. Solid binary systems of the drug with the selected CD were then prepared and adequately characterized by DSC and XRPD analyses. The obtained solutions were then subjected to storage and photo stability studies to evaluate the effect of the CD complexation on drug stability. Finally, the improved palatability of the drug CD complexes with respect to the drug alone was assessed by the use of the electronic tongue, which proved to be an effective tool in the early screening of the taste of pharmaceutical formulations and was particularly useful in expediting the development of pediatric dosage forms [47,48,49,50].

## 2. Materials and Methods

### 2.1. Materials

Propranolol.HCl (PPN) was purchased by Fagron Italia (Quarto Inferiore, Bologna, Italy). Natural cyclodextrins (αCD, βCD, γCD) were from SIGMA (St. Louis, MO, USA); randomly chemically modified hydroxypropyl-α-CD (HPαCD) (Cavasol^®^W6HP), hydroxypropyl-β-CD (HPβCD) (Cavasol^®^W7 HP), and hydroxypropyl-γ-CD (HPγCD) (Cavasol^®^W8 HP), all with an average molar substitution degree per anhydroglucose unit of 0.6, were from Wacker Chemie AG (Burghausen, Germany). All other chemicals were of analytical reagent grade. Purified water obtained by reverse osmosis (Elix^®^, Millipore, Baltimore, MD, USA) was used throughout the study.

### 2.2. Continuous Variation Method

The continuous variation method, also known as Job’s method [51,52], was used to evaluate the complexing ability of the drug of the various natural and derivative CDs considered and the stoichiometry of the respective complexes. Stock solutions of equimolecular concentrations of PPN (guest) and each CD (host) were prepared. Different volumes of such guest and host solutions were then mixed so that the total concentration ([PPN] + [CD]) remained constant (0.05 mM), while the guest molar fraction (X_PPN_ = [PPN]/([PPN] + [CD])), as well as the host molar fraction (X_CD_ = [CD]/([PPN] + [CD])), varied in the range of 0–1. The solutions were then suitably diluted, and their UV absorbance at 289.6 nm (λ_max_ of PPN) was measured (Shimadzu 1601 UV/Vis spectrophotometer, Tokyo, Japan) and compared with the corresponding solutions containing the same drug concentration but in the absence of CD. Graphs (Job plots) were then obtained by plotting Δ_abs_ (i.e., the difference between the PPN absorbance measured in the presence or absence of CD) vs. X_CD_ (i.e., the CD molar fraction). The value of X_CD_, for which the plot presents its maximum, gives the stoichiometry of the inclusion complex; moreover, the greater the maximum value of the curve, the stronger the stability of the complex.

### 2.3. Preparation of Drug-CD Solid Systems

Solid drug CD binary systems at both 1:1 and 1:2 molar ratios were prepared by co-grinding (GR) the respective physical mixtures in a high-energy vibrational micro-mill (Mixer Mill MM 200 Retsch, GmbH, Düsseldorf, Germany) for 30 min at a frequency of 24 Hz. Physical mixtures (PMs) were prepared by tumble mixing the respective simple components for 15 min (75–150 µm sieve granulometric fraction) at 1:1 or 1:2 molar ratios.

### 2.4. Differential Scanning Calorimetry (DSC)

DSC analyses of the single components and the different drug CD combinations were carried out using a MettlerTA4000 Star^e^ system equipped with a DSC 25 cell (Mettler Toledo, Greifensee, Switzerland). Exactly weighed samples (5–10 mg, Mettler MX5 Microbalance, Mettler Toledo, Greifensee, Switzerland) were scanned at 10 °C/min in pierced Al pans in the 30–200 °C temperature range under static air. The instrument was calibrated using Indium as a standard (99.98% purity; melting point 156.61 °C; fusion enthalpy 28.71 J g^−1^). The results are the mean of three separate experiments. The relative degree of crystallinity of PPN in the samples was estimated by the following equation:

(1) $$\mathrm{RDC}\%=\frac{{∆H}_{sample}}{{∆H}_{st}}\times 100$$

where ΔH_sample_ and ΔH_st_ are the heats of fusion of PPN measured in the samples and the starting pure untreated PPN, respectively.

### 2.5. X-ray Powder Diffractometry (XRPD)

X-ray powder diffraction patterns of the single components and the different PPN-CD combinations were recorded at ambient temperature using a Bruker D8 advance apparatus (Bruker Corp., Billerica, MA, USA) with a Cu Kα radiation and a graphite crystal monochromator at 40 mV voltage and 55 mA current over a 5–35° 2Θ range at a scan rate of 0.05 s^−1^.

### 2.6. Chemical and Physical Stability Studies during Storage

Aqueous solutions at 0.2% *w*/*v* drug, prepared starting from pure drug or drug:HPβCD PM and GR products at both 1:1 and 1:2 molar ratios, were stored for six months at 25 °C (room temperature), 4 °C, and 40 °C, and were wrapped in Al foils and checked every 30 days for drug content (by UV analysis at 289.6 nm, as described above) and pH (pH-meter Crison Basic 20, Crison Instruments, Modena, Italy) and subjected to visual inspection to evaluate limpidity and eventual changes of color and the formation of precipitates or mold. Samples were examined in triplicate.

Storage stability studies were also performed, for comparison purposes, on three different PPN aqueous solutions extemporaneously prepared according to the instructions of the SIFO (Società Italiana Farmacie Ospedaliere) galenic handbook [28], as is commonly performed in Italian hospital pharmacies. The composition of the prepared solutions is shown in Table 1.

### 2.7. Photo Stability Studies

Aqueous solutions at 0.2% *w*/*v* drug, prepared starting from pure drug or drug:HPβCD physical mixtures and GR products at both 1:1 and 1:2 molar ratios, were exposed to UV(A)–UV(B) radiations as solar light simulators. Briefly, samples of the solutions were put into quartz cells (1 cm path length), closed, and exposed to UV(A)–UV(B) radiations (xenon-arc lamp) for increasing irradiation times from 15 min to 300 min [42]. Immediately after irradiation, the solutions were subjected to UV analysis at 289.6 nm to determine PPN residual content.

### 2.8. Electronic Tongue Analysis

The effectiveness of propranolol unpleasant taste masking, obtained by complexation with HPβCD, was evaluated using the commercially available e-tongue Taste Sensing System SA 402B (Intelligent Sensor Technology- INSENT, Atsugi, Japan). The detecting part of the system consists of sensors whose surface is attached to artificial lipid membranes with different response properties to chemical compounds on the basis of their taste. In the present work, four detecting sensors and two reference electrodes were used, which were separated into two arrays according to the membrane charge: negative (AC0 and AN0 sensors) and positive (C00 and AE1 sensors). The detecting sensors were specific for the evaluation of bitterness (AC0—bitterness 1 and AN0—bitterness 2) aftertaste bitterness (C00) and aftertaste astringency (AE1) in pharmaceutical formulations [47]. E-tongue measurements were performed on solutions containing 1:1 or 1:2 mol:mol drug:HPβCD complexes and solutions containing pure drug (0.2% *w*/*v*) and pure HPβCD in the amounts used to obtain 1:1 or 1:2 mol:mol complexes. Each sample was evaluated two times, and the averages of the sensor outputs were converted to “taste intensity” values by multiplying sensor outputs for appropriate coefficients based on the Weber–Fechner law [48]. E-tongue data were analyzed by principal component analysis (PCA) [49] that was performed in a covariance matrix at a scale that was the same for all the e-tongue sensors [50]. E-tongue data were elaborated in the Minitab 17 software package (v. 1.0, Minitab Inc., State College, PA, USA).

## 3. Results

### 3.1. Selection of the Most Suitable CD for PPN Complexation

**Figure 1:**
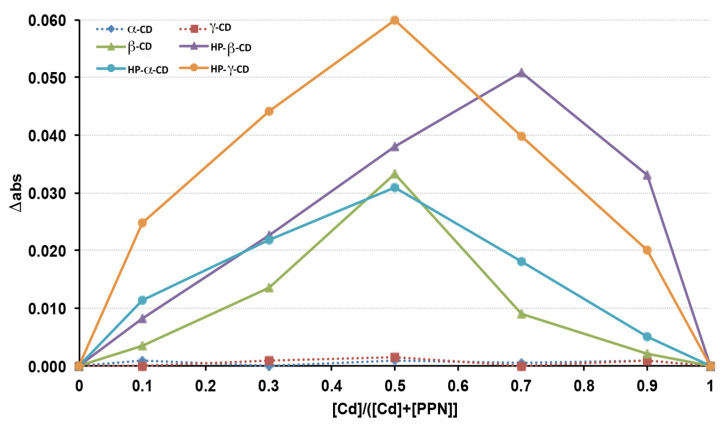
Continuous variation plots of PPN complexation with natural and HP derivative CDs.

A preliminary screening was performed to evaluate the complex ability of the drug of the three natural CDs (αCD, βCD, and γCD) and their hydroxypropyl-derivatives to determine the binding stoichiometry of their respective complexes. With this aim, the continuous variation method (also known as Job’s method [51,52]), was applied. The Job plots, obtained by graphing Δ_abs_ (the difference between PPN absorbance in the presence of CD) vs. the CD molar fraction (X_CD_), are shown in Figure 1. As can be seen, among the natural CDs, only βCD was able to complex the drug, giving rise to an evident variation of its UV absorbance, while no drug absorbance variations were observed in the presence of αCD and γCD with respect to the corresponding solutions containing the drug alone, suggesting the absence of drug complexation. These results were in agreement with Castronuovo et al. [53], who proved that PPN complexation with CDs happens by the inclusion of its naphtyl group into the CD cavity and that βCd enabled the best drug fit; on the contrary, the αCD cavity was too small to allow the PPN naphtyl group accommodation, and the γCD cavity was too large to lead to stable interactions with the guest molecule.

On the other hand, the complex formation happened with all the examined CD derivatives (HPαCD, HPβCD, and HPγCD), where bell-shaped curves were obtained. In particular, based on the higher intensity of the drug absorbance variation, it can be deduced that the most stable complexes were obtained with HPγCD and HPβCD. The improved complexing ability of PPN showed by all the considered CD derivatives with respect to their corresponding natural CDs can be attributed to the presence of the hydroxypropyl groups, which improved their hosting performance by enhancing their possible interactions with the guest molecule. In fact, the presence of the substituents amplified the hydrophobic region of the macromolecule by capping the edge of the cavity and enhancing the substrate binding ability via a hydrophobic effect [54,55,56].

In all cases, the Δ_abs_ maximum corresponded to a CD mole fraction of 0.5, indicating the formation of a complex with 1:1 drug:CD stoichiometry, except in the case of HPβCD, where the Δ_abs_ maximum was observed for a CD mole fraction of 0.7, suggesting that both 1:1 and 1:2 drug:CD complexes were simultaneously present in the solution. Based on the obtained results, HPβCD was chosen, not only for its good complexing ability in the drug but also taking into consideration its consolidated and proven safe use in oral and parenteral formulations at relatively high doses [46,57]. Moreover, HPβCD is listed by the FDA as an approved inert material, and its monography is present in both the USP National Formulary and European Pharmacopoeia. Finally, it has been reported that the oral availability of HPβCD was less than 1% [46].

### 3.2. Preparation and Characterization of Drug-HPβCD Solid Systems

Drug-HPβCD solid systems were prepared by the dry co-grinding of their 1:1 or 1:2 mol:mol physical mixtures in a high-energy mill. This method was selected since mechanochemical activation by grinding is considered a quick, efficient, economical, sustainable, and eco-friendly solvent-free technique for obtaining CD inclusion complexes in the solid state [58]. This method often proved to be equally or even more efficient than other preparation methods, such as kneading, co-evaporation, or sealed heating [59,60], and sometimes co-lyophilization [55] despite the highly porous structure of the freeze-dried products, which contribute to improving their dissolution properties.

**Figure 2:**
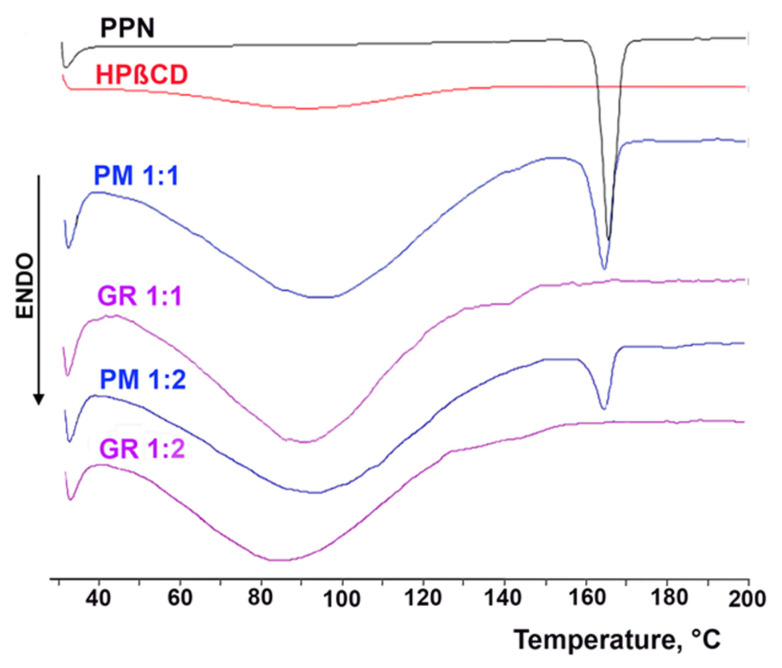
DSC curves of pure propranolol·HCl (PPN) and HPβCD and their 1:1 mol:mol and 1:2 mol:mol physical mixtures (PMs) and co-ground products (GRs).

The obtained co-ground (GR) systems were characterized in the solid state by DSC and PXRD analyses. The thermal curves of drug CD GR products are shown in Figure 2, together with the corresponding physical mixtures (PMs) and the pure components, and their relevant thermal parameters are presented in Table 2.

The DSC curve of PPN was typical of a pure crystalline anhydrous substance and was characterized by a flat profile followed by an intense and sharp endothermic effect, peaking at 163.92 °C, which is associated with the drug melting. The thermal profile of the drug after 30 min of grinding at 24 Hz (i.e., the same mechanical treatment used for preparing drug CD GR products) did not change, and only showed a minimum variation of its fusion temperature (162.97 °C) and fusion enthalpy (110.27 vs. 121.91 J/g). This result proved that the drug was stable to the treatment, which did not cause its amorphization. The DSC curve of HPβCD was instead characteristic of an amorphous hydrated substance, presenting a broad endothermic band between 50 and 120 °C due to its dehydration. The thermal profile of PPN was substantially maintained in its 1:1 and 1:2 mol/mol PM with HPβCD, where only a small reduction in fusion enthalpy and a shift to a lower temperature of its melting peak was observed, which were both attributable to the effect of mixing with the excipient. On the contrary, the complete disappearance of the drug melting peak was observed in the case of both GR products. This evident change in the drug thermal behavior cannot be ascribed to the mechanic treatment, as demonstrated above, and can be assumed as proof of interactions between the components, thus confirming the ability of the co-grinding technique to induce effective drug CD solid-state interactions and drug amorphization.

**Figure 3:**
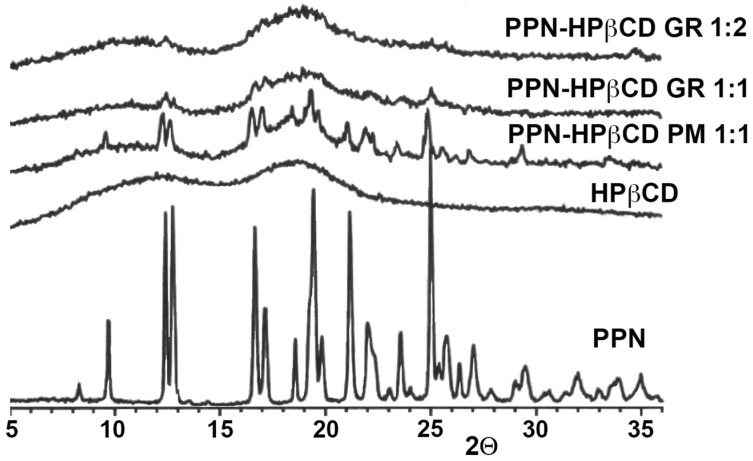
PXRD spectra of pure propranolol·HCl (PPN) and HPβCD and their 1:1 mol:mol physical mixture (PM) and 1:1 and 1:2 mol:mol co-ground products (GRs) with HPβCD.

Figure 3 shows the results of PXRD studies, which substantially confirmed DSC analyses. In fact, the PPN spectrum exhibited numerous sharp and intense peaks, indicative of its crystalline nature, while HPβCD presented the characteristic pattern of an amorphous substance. The typical crystallinity peaks of PPN were still detectable on the 1:1 and 1:2 mol/mol PMs, emerging from the amorphous halo pattern of HPβCD, while a total sample amorphization was found in both 1:1 and 1:2 mol/mol GR products. Moreover, this result allowed us to definitely exclude amorphization phenomena due to the sample heating during the DSC scan.

### 3.3. Photo Stability Studies

**Figure 4:**
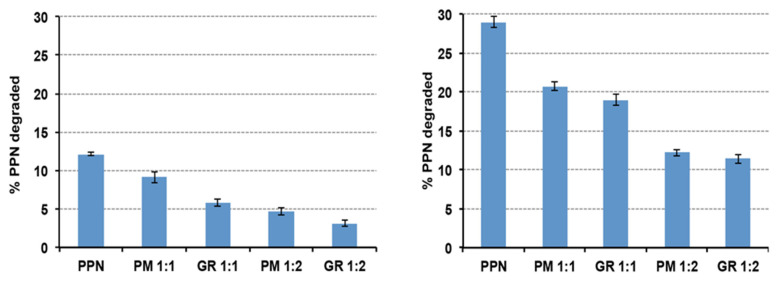
% PPN degraded after 1 h (**left**) or 5 h (**right**) of exposition to UV(A)–UV(B) radiations (xenon-arc lamp) of solutions prepared using PPN alone or as a 1:1 or 1:2 mol:mol physical mixture (PM) or co-ground (GR) with HPβCD.

The results of photo stability studies of the solutions at 0.2% *w*/*v* drug, prepared using the drug alone or as 1:1 or 1:2 mol:mol GR or simple PM with HPβCD, are shown in Figure 4, in terms of % drug degraded after 1 h or 5 h of exposition to UV(A)–UV(B) radiations.

As can be seen, a clear protective effect of HPβCD against the photodegradation of PPN was observed, with a reduction in the % degraded drug after 5 h of exposition to UV(A)–UV(B) radiations by more than 50% compared to the solution containing the drug alone. This result was in agreement with Ansolin et al. [45], who observed a reduction in the PPN photolysis rate thanks to its complexation with βCD as a consequence of the protection of the aromatic system of the drug by the CD nanocavity. We found that the observed protective effect of the drug was more evident during the whole exposition time for the 1:2 drug:HPβCD systems, i.e., when a higher amount of HPβCD was present in the solution. On the contrary, interestingly, while after 1 h of exposition, the HPβCD protective effect was more intense in the case of the GR product with respect to the simple PM, this difference was no more significant after 5 h of exposition. Probably, the initial higher protective efficacy obtained using the GR product can be attributed to the more intense interaction between the components, which was brought about by the mechanical treatment, which promoted complex formation. However, with time, the inclusion of complex formation in the solution also happened in the solution prepared using the PM, allowing it to reach a similar protective effect compared to the GR product.

The degradation constants of the drug in the various examined solutions were calculated using the linear equation regressions of the curves of the % residual drug as a function of time after 1 h and 5 h of exposure to UV(A)–UV(B) radiations (Table 3).

The final value of the degradation constant of PPN in the aqueous solution in the absence of HPβCD (−0.0891 min^−1^) was in agreement with Ansolin et al. [45] (−0.0860 min^−1^). As can be seen, a strong reduction in the drug degradation rate was obtained in the presence of HPβCD, and this effect was more evident in the samples containing higher amounts of CD (1:2 vs. 1:1 mol/mol systems). On the other hand, the lower degradation rate of the solutions containing the GR than the corresponding PM systems, observed during the first hour of exposure, was no more evident when considering the whole exposure period (5 h). As already hypothesized above, this was probably due to the gradual inclusion of complex formation in the solutions prepared with the simple PMs, which gave rise to the progressive decrease in their different behavior with respect to those prepared with the GR systems. The final % of drug stabilization of about 55% was in agreement with Ansolin et al. in the presence of βCD [45] (around 53–54%).

### 3.4. Storage Stability Studies

Stability studies are essential in the development of any drug formulation to evaluate the physical, chemical, and microbiological changes that can occur and could affect the quality, safety, and therapeutic efficacy of the drug, predict its stability during its permanence on the market, and assign it a shelf life. Storage stability studies were performed on aqueous solutions at 0.2% *w*/*v* drug alone or as 1:1 or 1:2 mol:mol PM or GR systems with HPβCD to be able to investigate the influence of the CD presence on the drug stability and exclude any possible destabilizing effects. In fact, although most studies revealed a favorable effect of CD complexation on drug stability [32,39,40,41,42,43,44,45], few reports about a catalytic effect of CDs on the drug degradation rate have been reported [61,62]. The stability studies were carried out at three different temperatures: refrigerated (4 °C), ambient (25 °C, climatic Zones I and II), and accelerated conditions (40 °C). All the samples were wrapped in Al foils to protect them from the light.

**Figure 5:**
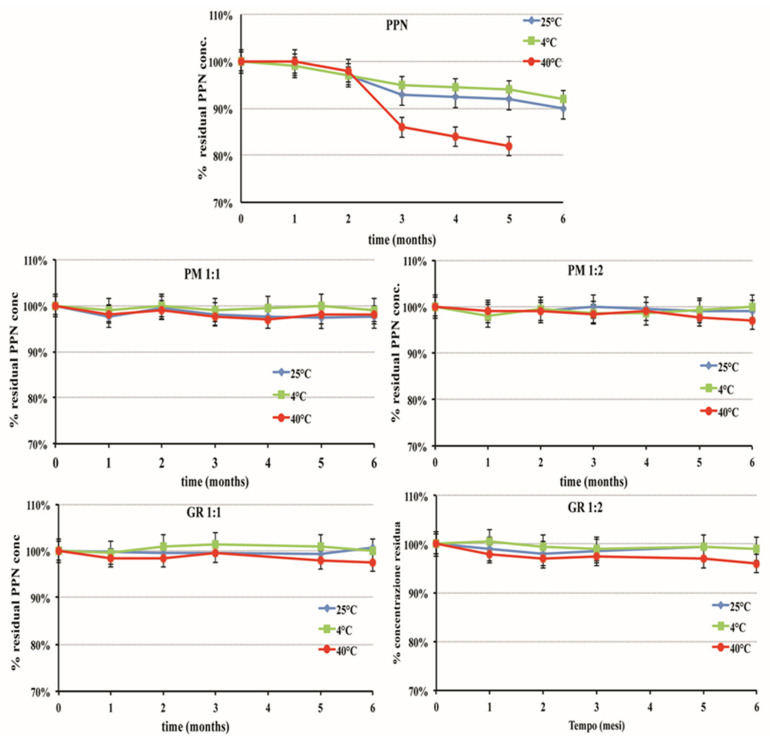
Percent of residual intact drug during storage stability studies at different temperatures of aqueous solutions at 0.2% *w*/*v* PPN alone or as 1:1 or 1:2 mol:mol PM or GR systems with HPβCD.

As can be seen in Figure 5, the drug concentration remained near the initial 100% value during the whole storage period (six months) in all the samples containing the HPβCD, where the concentration of intact PPN never dropped below 98% for the samples stored at ambient and refrigerated temperatures or 96% for those stored under accelerated conditions (40 °C). Since no significant changes over time occurred at the accelerated conditions, according to the ICH guidelines [63], an extrapolation of the retest period or shelf-life can be proposed, which can be up to twice the period covered by long-term data. Therefore, a shelf-life of 1 year at ambient temperature can be expected.

Moreover, these samples also remained unchanged in their organoleptic properties (limpidity and the absence of coloration) and pH (around 5.5) and did not show the formation of mold. A favorable influence of the CD presence on the microbiological stability of liquid formulations was previously observed in the case of SLN (solid lipid nanoparticles) and NLC (nanostructured lipid carriers) formulations of hydrochlorothiazide [64,65]. On the contrary, in the case of the solution containing the drug alone, a reduction in residual drug concentration was observed as early as three months of storage. This effect was more gradual in the case of samples stored at 4 °C or 25 °C, where the % of intact drug at the end of the storage period was almost 90%. On the contrary, it was clearly more evident for the samples stored at 40 °C, where a loss of about 15% was found after three months, until reaching almost 20% after six months. This was joined with a pH value increase, which reached up to 6.5 in the samples stored at 40 °C, and the formation of a needle-shaped crystalline precipitate that was revealed to be α-naphtole, as confirmed by DSC analysis (T_m_ 94.8 °C).

For comparative purposes, storage stability studies were also performed on three different PPN compounding liquid formulations (named A, B, and C), which were prepared according to the SIFO galenic handbook (see Table 1 for their composition). All these formulations showed problems of poor physical and/or microbiologic stability. In fact, solution A showed the appearance of mold after only one month of storage at room temperature (25 °C) and after two months at 4 °C. On the other hand, the higher sucrose concentration present in formulations B and C protected them against mold development but gave rise to other drawbacks, causing the brown coloration of solution B after two months at 40 °C and the appearance of the sugar crystallization phenomena after two months at 4 °C in solution C. No appreciable reduction in intact drug concentration was instead observed in up to three months of storage. The good chemical stability of these solutions should be mainly attributed to their low pH value (around 3), which, however, could give rise to problems of poor tolerability, especially in prolonged treatments. Moreover, the presence of sucrose makes them unsuitable for diabetic patients.

### 3.5. Palatability Studies

The CD’s ability to mask the unpleasant taste of drugs has been proved by several authors [32,33,34,35,36,37,38] and has been mainly attributed to the complete or partial inclusion of the drug molecules into the nanocavity of the CD molecules, with a consequent reduction in their interactions with the taste buds. An appropriate taste evaluation method must, nevertheless, be used to determine the actual efficacy of this taste-masking strategy since good palatability is a crucial factor in determining medicine acceptance and improving patient compliance, particularly in the pediatric field.

The human taste panel test is considered the best method to evaluate the overall palatability and acceptability of a drug formulation by patients, but its use is limited by a series of logistic, economic, ethical, and safety concerns, particularly at the level of pre-formulation or preclinical phases of drug development. Then, to overcome these drawbacks, various non-human tools for taste evaluation have been developed, including the use of the electronic tongue (e-tongue) [66]. This device proved to be very effective, particularly for drug bitterness evaluation [67,68], and showed a good correlation with the human test [66]. In this study, the e-tongue was selected to evaluate the taste of the developed HPβCD-based formulations, and the collected data were first processed by PCA, allowing the visualization of the relationships between the samples (score plot) and variables (loading plot).

**Figure 6:**
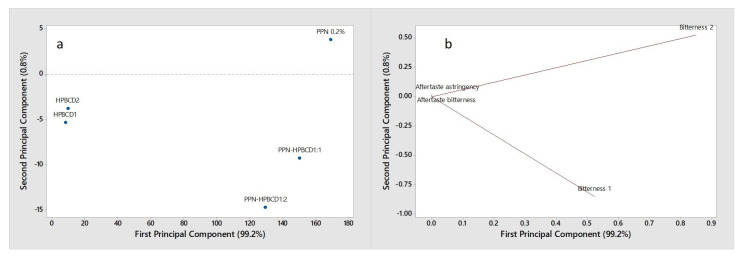
PCA score plot (**a**) and loading plot (**b**) of the e-tongue data collected on HPβCD, PPN, and PPN-HPβCD 1:1 and 1:2 mol/mol GR systems.

In Figure 6, the score plot and the loading in the plane defined by the first two principal components (PC1 and PC2) explaining the total variability (100% of the total variance) are shown.

Considering the score plot (Figure 6a), a clear separation of the samples is evident along the PC1 (99.2% explained variance). In particular, the pure HPβCD samples, in the amount corresponding to 1:1 mol:mol (HPβCD1) and 1:2 mol:mol (HPβCD2), located at the left of PC1, are well-discriminated by PNN and 1:1 and 1:2 drug:HPβCD systems, which are located on the opposite side of the plot. By the loading plot (Figure 6b), it can be observed that the two bitterness sensors (bitterness 1 and bitterness 2) characterize the analyzed samples and are significant in their discrimination, while the aftertaste bitterness and aftertaste astringency are not relevant.

**Figure 7:**
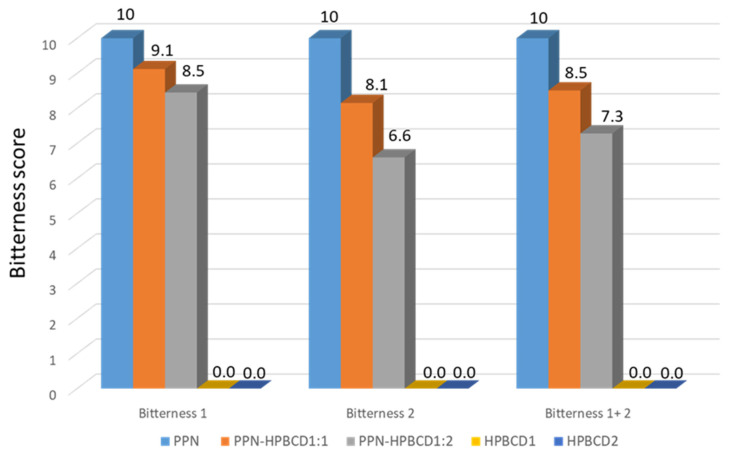
Bar graph of the e-tongue-assessed bitterness scores of HPβCD, PPN, and the PPN-HPβCD 1:1 and 1:2 mol/mol GR systems.

Since the bitter taste is the only relevant one, considering the bitter intensity of the samples measured by e-tongue, the bitterness scores were evaluated and reported in a bar graph (Figure 7).

The bitterness scores of the formulations were calculated for bitterness 1, bitterness 2, and total bitterness (bitterness 1 + 2) by setting the bitterness intensity of the HPβCD samples (HPβCD1 and HPβCD2) to 0 and the bitterness intensity of PPN to 10. Considering the 1:1 PPN-HPβCD formulation, the total bitterness score (bitterness 1 + 2) is equal to 8.5, corresponding to a bitterness reduction of 15% with respect to PPN; for the 1:2 PPN-HPβCD formulation, the total bitterness score (bitterness 1 + 2) is equal to 7.3, corresponding to a bitterness reduction of about 30% with respect to PPN.

## 4. Conclusions

A safe, stable, and palatable liquid formulation of PPN that is suitable for oral administration in pediatrics has been developed by exploiting the drug complexation with HPβCD to overcome its problems of instability to light, stability only at acidic pH (around 3), and unpleasant bitter taste.

Drug HPβCD physical mixtures or co-ground systems at 1:1 or 1:2 mol:mol ratios were used to prepare 0.2% *w*/*v* drug solutions.

Photo stability studies showed a clear protective effect of HPβCD, which enabled a reduction of up to 75% or 55% of the drug degradation rate after 1 or 5 h of exposure to UV radiation, respectively.

Storage stability studies performed during 6 months at 4, 25, and 40 °C proved the maintenance of unchanged physical–chemical properties and almost constant drug concentration under accelerated conditions (40 °C) despite the less aggressive pH (around 5.5) of the solutions with respect to the lower pH required to assure drug stability (pH 3).

The palatability test with the electronic tongue evidenced the taste-masking properties of HPβCD, which allowed a reduction of drug bitterness by up to 30%.

In conclusion, taking these results together, it appears evident that the developed solutions based on the combination of PPN with HPβCD can represent a valid alternative to the extemporaneous compounding solutions. In fact, they not only showed greater physical and microbiologic stability and better tolerability (pH 5.5 rather than 3.0, commonly used to enhance drug stability), but also made it possible to avoid the need to use sucrose or flavoring agents (and their solvents, such as propylene glycol), which are usually added to improve palatability but are potentially harmful for pediatric patients.

## Figures and Tables

**Table 1:** Quali-quantitative composition of 100 mL of the examined PPN solutions extemporaneously prepared according to the SIFO (Società Italiana Farmacie Ospedaliere) galenic handbook [28].

Components	Solution A	Solution B	Solution C
PPN	0.2 g	1.0 g	0.1 g
Sucrose	25 g	-	-
Citric acid anhydrous	0.64 g	-	-
Na citrate dihydrate	0.20 g	-	-
Raspberry flavour	0.1 mL	0.05 mL	0.05 mL
Citric acid monohydrate	-	0.60 g	0.60 g
Simple syrup FU	-	q.b. to 100 mL	q.b. to 100 mL
Water for injections	q.b. to 100 mL	q.b. to solubilize PPN	q.b. to solubilize PPN

**Table 2:** Melting temperature (T_m_), fusion enthalpy (ΔH), and % residual drug crystallinity (RDC) of propranolol·HCl (PPN) and its 1:1 and 1:2 mol:mol physical mixtures (PMs) and co-ground (GR) with HPβCD.

Sample	T_m_ (°C)	ΔH_fus_ (J/g)	%RDC
PPN	163.92	121.91	100.0
PPN ground 15′ at 24 Hz	162.22	112.59	92.30
PPN ground 30 at 24 Hz	162.81	110.27	90.45
PPN-HPβCD PM 1:1	164.20	90.20	73.98
PPN-HPβCD PM 1:2	163.80	85.52	70.15
PPN-HPβCD GR 1:1	-	-	-
PPN-HPβCD GR 1:2	-	-	-

**Table 3:** Degradation constants of propranolol·HCl (PPN) in the aqueous solutions containing the free drug or its 1:1 or 1:2 mol/mol physical mixture (PM) or co-ground product (GR) with HPβCD calculated after 1 h and 5 h of exposure to UV(A)–UV(B) radiations and % stabilization.

Solution Sample	K_0–1 h_ (%/min)	% Stabilization	K_0–5 h_ (%/min)	% Stabilization
PPN	−0.2014		−0.0891	
PPN: HPβCD 1:1 PM	−0.1517	24.7	−0.0626	29.7
PPN: HPβCD 1:2 PM	−0.0956	52.5	−0.0402	54.9
PPN: HPβCD 1:1 GR	−0.0775	61.5	−0.0594	34.4
PPN: HPβCD 1:2 GR	−0.0541	73.1	−0.0398	55.3

## Data Availability

Not applicable.
